# Special Issue “Emerging Viruses 2021: Surveillance, Prevention, Evolution and Control”

**DOI:** 10.3390/v14040815

**Published:** 2022-04-15

**Authors:** Fabrício Souza Campos, Maité Freitas Silva Vaslin, Luciana Barros de Arruda

**Affiliations:** 1 Laboratório de Bioinformática & Biotecnologia, Campus de Gurupi, Universidade Federal do Tocantins, Gurupi 77402-970, TO, Brazil; 2 Departamento de Virologia, Instituto de Microbiologia Paulo de Góes, Universidade Federal do Rio de Janeiro, Rio de Janeiro 21941-902, RJ, Brazil

Virus replication frequently results in the accumulation, re-assortment and re-combination of mutations, which contributes to their rapid adaptation to environmental changes and often advances the emergence of new virus variants or species. These features, in addition to globally distributed anthropogenic activities and human dispersal, have resulted in an increased frequency of outbreaks, epidemics and pandemics. The emergence and re-emergence of novel pathogens presume complex and changeable host–pathogen interactions and co-evolution, challenging public health and agricultural systems for the development of cost-effective diagnostic methods and therapeutic and prevention strategies, besides maintaining efficient epidemiological surveillance. Thus, this Special Issue of Viruses is a collection of high-quality science and appropriate monitoring in the face of the challenges experienced globally with the COVID-19 pandemic. We are proud to have received a total of 24 manuscript submissions with an acceptance rate of 75.0% (18 papers) related to virus surveillance and evolution, diagnosis, pathogenesis, clinical aspects, host immunity and treatment, involving human and animal viruses.

In the face of the current pandemic, many of the published manuscripts are related to SARS-CoV-2. Moreira et al. [1] conducted a genomic epidemiology study focused on characterizing the dissemination of SARS-CoV-2 B.1.1.7 lineage in Brazil. At the time the manuscript was published, this lineage was present in ten Brazilian states and an increased frequency with a higher number of cases was expected in the country, considering the reproduction number estimated from UK.

In the same sense, Francisco Junior et al. [2] carried out a retrospective genomic epidemiology analysis of the SARS-CoV-2 pandemic in the Rio de Janeiro state, Brazil. The data were obtained from GISAID, corresponding to 1927 new sequenced genomes, which were sampled from March to June 2021. Results showed the importance of viral supercarriers in the virus dispersion and the presence of two new variants (P.5 and P.1.2). Thus, the findings provided important lessons learned from the different epidemiological aspects of the SARS-CoV-2 dynamic in Rio de Janeiro, Brazil.

Lesbon et al. 2021 [3] identified mutations in the nucleocapsid (N) gene that affected the golden standard test, the RT-qPCR assay. For this, the authors sequenced SARS-CoV-2 genomes from 17 positive samples, from which RNA-dependent RNA polymerase and envelope genes, but not N gene, had been detected. They identified three different mutations affecting N detection: an 18-nucleotide deletion (Del28877–28894), a substitution of GGG for AAC (28881–28883) and a frameshift mutation caused by the deletion (Del28877–28878). Thus, the authors point out that continuous monitoring and characterization of mutations that affect the hybridization sites of primers and probes by genomic surveillance programs are necessary to maintain the effectiveness of the COVID-19 diagnosis. Kubik et al. [4] investigated the emergence patterns of spike mutations in sequences deposited in local and global databases to identify mutational hotspots across phylogenies. The authors found diverse substitutions at a critical residue, positioned in the N-terminal domain of the spike protein, which were repeatedly observed independent of phylogenetic and geographical contexts. The increased frequency of recruitment of these mutations suggests that they might contribute to viral evolution and could impact on the evasion of neutralizing antibodies. Using a different approach, Miller et al. [5] used computational and structural analyses of over 50 mAb/nanobody-RBD/NTD epitope–paratope interfaces to capture the role of each RBD and NTD spike protein residue interacting with polyclonal antibodies. These data generated an antigenic space map, which was integrated with experimental datasets describing genetic, structural and functional constraints on mutation to predict potential antigenic drift sites, with higher risk of antibody evasion.

In Colson and Raoult’s [6] brief report, the number of SARS-CoV-2 genome sequences obtained from 135 countries on the 5 continents until January 2021 were correlated with the number of SARS-CoV-2-diagnosed cases, number of SARS-CoV-2-associated deaths, population size, life expectancy, gross domestic product (GDP) per capita and human development index per country, based on the GISAID database and “Our World in Data” website, respectively. When considering the 19 countries for which the number of genomes per 100 deaths was >100, great discrepancies were detected among wealthy and developing countries, with some of the developed ones showing similar or lower numbers of sequences in relation to countries with lower development indexes. The authors emphasize that a broad-scale SARS-CoV-2 genomic surveillance should be a priority for all developed countries that can afford and manage it.

Beck et al. [7] developed a novel semi-supervised pipeline for automated gene, protein and functional domain annotation of SARS-CoV-2 genomes, aiming to overcome the limitations imposed by the reference-guided approach due to the continuing virus evolution rate. For this, the authors analyzed 66,000 SARS-CoV-2 genome sequences and identified the comprehensive set of known proteins with 98.5% set membership accuracy and 99.1% accuracy in length prediction, yielding increased genome and protein annotations, in comparison to other published tools.

Regarding virus treatment, Chan et al. [8] screened a small library of FDA-approved compounds, using a pseudovirus system, and identified structurally related compounds that inhibited the early entry steps of the SARS-CoV and SARS-CoV-2 infectious cycle in vitro. The authors also discussed the prospects of repurposing these drugs for treating current and future coronavirus outbreaks.

In the opinion section, Moelling [9] discussed the idea that SARS-CoV-2 replication for longer periods in immunocompromised patients may allow a higher mutation rate and promote “within-host evolution”. The author suggests that some of the viruses could become founders, further contributing to “between-host evolution”, and reinforce that immunosuppressed patients should be supervised by healthcare workers to avoid new variants with pandemic potential.

The host immune response profile during human virus infections was another important topic discussed in this issue. Zheng et al. [10] investigated the circulating levels of inflammatory cytokines in 140 patients diagnosed with human adenovirus (HAdV) who presented different illness severities. The authors observed that the number of circulating lymphocytes were reduced in the severe patients, whereas IL-6, IL-10 and IFN-γ levels were increased when compared with mild patients or healthy controls. In vitro cultures suggested the increased frequency of activated TCD8^+^ cells, which might be related to disease severity.

Netto et al. [11] conducted a cross-sectional study to evaluate the frequency of hypovitaminosis D and its correlation with the immune response in HTLV-infected patients. A significantly higher prevalence of hypovitaminosis D was detected in patients with HTLV-1-associated myelopathy/tropical spastic paraparesis (HAM/TSP), in comparison to HTLV asymptomatic carriers or controls. HAM/TSP patients also presented higher levels of IL-6 and IFN-γ than asymptomatic carriers. In addition, a negative correlation between TNF-α and vitamin D was observed in the patients with HAM/TSP, suggesting that the role of hypovitaminosis D in HAM/TSP pathogenesis should be evaluated further.

Not only human viruses were addressed in this special issue Influenza virus infection is a major cause of the severe acute respiratory syndrome, and birds are one of the main sources of those viruses, potentially contributing to the emergence of new future pandemics. Thus, it is essential to monitor wild birds to follow the evolution of influenza viruses. Trinh et al. [12] investigated the genome sequence, as well as the in vitro and in vivo replication of a new H7N3 avian influenza virus isolated from spot-billed ducks in South Korea in 2019. Molecular analyses indicated that this isolate was a typical low-pathogenicity avian influenza virus (LPAIV) and revealed its close identity with others, such as Influenza A from Shanghai, Egypt and South Korea. It was also pointed out that these new Korea H7N3 viruses, carrying multiple potential mutations, have the potential to become highly pathogenic and develop the ability to infect humans, emphasizing the need for routine surveillance of avian influenza viruses in wild birds.

An outbreak of another important virus pathogen in birds—the Newcastle disease virus (NDV)—was reported by Thomazelli et al. [13], after the surveillance of feral pigeons in São Paulo city, Brazil. Affected birds displayed neurological signs, with NDV antigen staining detected in the brains. Additionally, hemorrhagic events were observed in different tissues and the infiltration of mononuclear inflammatory cells was observed in the brain, kidney, proventriculus, heart and spleen by histopathology analyses. Part of the tested samples were positive for the Newcastle disease virus by RT-qPCR test, and one virus isolate was fully sequenced. Phylogenetic analysis grouped the detected isolate with other viruses from subgenotype VI.2.1.2, class II. Due to the zoonotic risk of NDV, virus surveillance in feral pigeons should also be systematically performed in urban areas.

Another important avian pathogen, the retrovirus reticuloendotheliosis virus (REV), associated with immunosuppression, anemia, proventriculitis, neoplasia and runting stunting syndrome in poultry farms, was also the focus of a study published in this Special Issue. Chacón et al. [14] characterized the complete genome of two new REV strains detected in Brazilian chickens with multiple viral coinfections. Phylogenetic analysis grouped these Brazilian strains into REV subtype 3, closely to USA REV and fowlpox virus (FWPV)-related strains. Most of the studied farms (90%) showed the presence of REV strains in chickens co-infected with several other virus, suggesting that REV may be acting as an immunosuppressive potentially activating the emergence and progression of multiple infectious diseases. This is the first report of REV in South America. The same group, Chacón et al. [15], identified the presence of a novel clade E aviapoxvirus in Southeast Brazil, after investigating outbreaks of possible avipoxvirus in commercial breeder flocks of avipoxvirus-vaccinated chickens. Avian poxviruses affect wild and domestic birds, producing proliferative lesions on the nonfeathered parts of the body (the cutaneous form) or necrotic lesions in the upper respiratory tract (the diphtheritic form). The disease is usually controlled by live attenuated vaccines. Nevertheless, bollinger bodies and poxvirus-like particles were identified in the clinical samples by light and transmission electron microscopy and the sequencing of PCR-amplified fragments showed that the outbreaks were caused by clade E avipoxvirus. This report shows that this new pathogen is spreading in the Brazilian poultry industry and highlights the need for permanent vigilance of avian poxviruses, including improving the sanitary and vaccination programs.

Camelpox virus (CMLV), a poxvirus that affects camelids, usually associated with season outbreaks in the Old World, was studied by Joseph et al. [16]. The group characterized an outbreak of a systemic form of camelpox in seven dromedary camels from United Arab Emirates (UAE). Diagnostics were performed by histology, virus isolation and PCR. Phylogenetic analysis indicated a single phylogenetic group, and the UAE isolate showed closest similarity with CMLV isolates from Israel. Interestingly, despite the closely related sequences between the isolates from those two countries, disease manifestation reported in these outbreaks was vastly different, indicating that CMLV virulence is not only determined by genetic features, but may also depend on aspects of the host (e.g., age, overall fitness), management and the environment.

Finally, Arruda et al. [17] described the Brazilian Society for Virology’s (SBV) annual meeting in 2020, which took place online for the first time in history. Despite the challenge of the new format, the Brazilian society board and collaborators were successful in virtually congregating more than 921 attendees, which was the greatest SBV participant number ever reached. Seminal talks from prominent national and international researchers were presented every night for a week, and included discussions about environmental, basic, animal, human, plant and invertebrate virology. A special roundtable, made up of some of the greatest Brazilian virologists, exclusively debated new data and perspectives regarding COVID-19. Women scientists were very well represented in another special roundtable called “Young Women Inspiring Research”, which was one of the most viewed and commented section during the meeting, given the extraordinary quality of the presented work. Next, Vaslin et al. [18] described the 32nd annual meeting which occurred in 2021 and was once again an online meeting. Similar to the 2020 meeting, the number of attendees was high, with considerable participation from undergraduate, graduate and postdoc students. Distinguished scientists from different countries offered high-quality conferences, and oral presentation sessions were presented by young scientists showing their newest research results. For almost five hours a day across five whole days, attendees discussed high-quality science related to all areas of virology. Even with the difficulties imposed by another pandemic year, the 32nd SBV annual meeting achieved its most important goal—to inspire young scientists and discuss high-quality virology research.

## References

[1] Moreira F.R.R., Bonfim D.M., Zauli D.A.G., Silva J.P., Lima A.B., Malta F.S.V., Ferreira A.C.S., Pardini V.C., Magalhães W.C.S., Queiroz D.C. (2021). Epidemic Spread of SARS-CoV-2 Lineage B.1.1.7 in Brazil. Viruses.

[2] Francisco Junior R.D.S., Lamarca A.P., de Almeida L.G.P., Cavalcante L., Machado D.T., Martins Y., Brustolini O., Gerber A.L., Guimarães A.P.C., Gonçalves R.B. (2021). Turnover of SARS-CoV-2 Lineages Shaped the Pandemic and Enabled the Emergence of New Variants in the State of Rio de Janeiro, Brazil. Viruses.

[3] Lesbon J.C.C., Poleti M.D., de Mattos Oliveira E.C., Patané J.S.L., Clemente L.G., Viala V.L., Ribeiro G., Giovanetti M., de Alcantara L.C.J., de Lima L.P.O. (2021). Nucleocapsid (N) Gene Mutations of SARS-CoV-2 Can Affect Real-Time RT-PCR Diagnostic and Impact False-Negative Results. Viruses.

[4] Kubik S., Arrigo N., Bonet J., Xu Z. (2021). Mutational Hotspot in the SARS-CoV-2 Spike Protein N-Terminal Domain Conferring Immune Escape Potential. Viruses.

[5] Miller N.L., Clark T., Raman R., Sasisekharan R. (2021). An Antigenic Space Framework for Understanding Antibody Escape of SARS-CoV-2 Variants. Viruses.

[6] Colson P., Raoult D. (2021). Global Discrepancies between Numbers of Available SARS-CoV-2 Genomes and Human Development Indexes at Country Scales. Viruses.

[7] Beck K.L., Seabolt E., Agarwal A., Nayar G., Bianco S., Krishnareddy H., Ngo T.A., Kunitomi M., Mukherjee V., Kaufman J.H. (2021). Semi-Supervised Pipeline for Autonomous Annotation of SARS-CoV-2 Genomes. Viruses.

[8] Chan S.-W., Shafi T., Ford R.C. (2021). Kite-Shaped Molecules Block SARS-CoV-2 Cell Entry at a Post-Attachment Step. Viruses.

[9] Moelling K. (2021). Within-Host and Between-Host Evolution in SARS-CoV-2—New Variant’s Source. Viruses.

[10] Zheng R., Li Y., Chen D., Su J., Han N., Chen H., Ning Z., Xiao M., Zhao M., Zhu B. (2021). Changes of Host Immunity Mediated by IFN-γ^+^ CD8^+^ T Cells in Children with Adenovirus Pneumonia in Different Severity of Illness. Viruses.

[11] Netto E.C., Silva A.C., Pedroso C., Brites C. (2021). Hypovitaminosis D Is Associated with Higher Levels of Inflammatory Cytokines and with HAM/TSP in HTLV-Infected Patients. Viruses.

[12] Trinh T.-T.T., Tiwari I., Durairaj K., Duong B.T., Nguyen A.T.V., Tuong H.T., Hoang V.T., Than D.D., Nam S., Yeo S.-J. (2021). Genetic Characterization and Pathogenesis of Avian Influenza Virus H7N3 Isolated from Spot-Billed Ducks in South Korea, Early 2019. Viruses.

[13] Thomazelli L.M., Sinhorini J.A., Oliveira D.B.L., Knöbl T., Bosqueiro T.C.M., Sano E., Costa G.C.V., Monteiro C., Dorlass E.G., Utecht N. (2021). An Outbreak in Pigeons Caused by the Subgenotype VI.2.1.2 of Newcastle Disease Virus in Brazil. Viruses.

[14] Chacón R.D., Sedano-Herrera B., Alfaro-Espinoza E., Quispe W.U., Liñan-Torres A., De la Torre D., de Oliveira A., Astolfi-Ferreira C., Ferreira A.J.P. (2022). Complete Genome Characterization of Reticuloendotheliosis Virus Detected in Chickens with Multiple Viral Coinfections. Viruses.

[15] Chacón R.D., Astolfi-Ferreira C., Pereira P.C., Assayag M.S., Campos-Salazar A.B., De la Torre D., de Sá L.R.M., de Almeida S.R.Y., Ricci R.E.G., Ferreira A.J.P. (2022). Outbreaks of avipoxvirus clade E in vaccinated broiler breeder flocks with exacerbated beak injuries and sex differences in severity. Viruses.

[16] Joseph S., Kinne J., Nagy P., Juhász J., Barua R., Patteril N.A.G., Hoffmann D., Pfaff F., Hoffmann B., Wernery U. (2021). Outbreak of a Systemic Form of Camelpox in a Dromedary Herd (*Camelus dromedarius*) in the United Arab Emirates. Viruses.

[17] Arruda L.B., Campos F.S., Abrahão J.S., da Fonseca F.G., Araújo Junior J.P., Rosado Spilki F. (2021). 31st Brazilian Online Society for Virology (SBV) 2020 Annual Meeting. Viruses.

[18] Vaslin M.F.S., Leal A.A., Bueno L.M., Bittar C., de Souza G.F., Lourenço K., da Silva G.P.D., Guedes M.I.M.C., Proença-Módena J.L., Araújo Junior J.P. (2022). The 32nd Brazilian Society of Virology (SBV) 2021 Annual Meeting. Viruses.

